# Physical fitness tests as a requirement for physical performance improvement in officers in the military police of the state of Paraná, Brazil

**DOI:** 10.47626/1679-4435-2020-581

**Published:** 2021-03-03

**Authors:** Hallyne Bergamini Silva Caetano, Cristiano Israel-Caetano, José Francisco López-Gil, Rafael Gomes Sentone, Karyne Bergamini Silva Godoy, Fernando Renato Cavichiolli, Anderson Caetano Paulo

**Affiliations:** 1 Departamento de Educação Física, Universidade Tecnológica Federal do Paraná, Curitiba, PR, Brazil; 2 Departamento de Educação Física, Universidade Federal do Paraná, Curitiba, PR, Brazil; 3 Assessoria Técnica, Casa Militar, Polícia Militar do Paraná, Curitiba, PR, Brazil; 4 Departamento de Actividad Física y Deporte, Universidad de Murcia, Murcia Region, Spain

**Keywords:** aptitude tests, police, law enforcement, physical fitness, health planning

## Abstract

**Introduction:**

Physical fitness is crucial for the work of military police officers. Over time, officers show a substantial decrease in physical fitness. State military officers must undergo a physical fitness test every year, but prior to 2015, failure on this test had no impact on career advancement. State Law No. 18.659/2015 included passage of the physical fitness test as a requirement for promotion for officers in the Paraná military police (Polícia Militar do Paraná).

**Objectives:**

To verify whether the obligation to undergo the physical fitness test had any effects on the physical performance of military police officers.

**Methods:**

The physical fitness tests results from 2016-2019 (n = 1705) were entered into an electronic spreadsheet and stratified by year. The spreadsheet included scores on individual tests (*shuttle run*, upper body and 12-minute run) as well as the sum total across all tests. Then, descriptive statistics, normality test, Kruskal-Wallis H test, and post-hoc comparisons were performed using the Mann-Whitney U test. with a significance level of p < 0.05.

**Results:**

Significant differences (p < 0.05) were observed on all variables in the physical fitness test when compared between the years of 2016, 2017, 2018 and 2019. Mean scores on the shuttle run (96.91 ± 7.54) and upper body tests (82.60 ± 24.81) were highest in 2019, while the mean score on the 12-minute run test was highest in 2017 (60.33 ± 28.38). The effect size of these differences (r) was small.

**Conclusions:**

The evidence suggests that the inclusion of the physical fitness test as a requirement for promotion can contribute to the improvement of physical performance in military police officers.

## INTRODUCTION

Police officers are at high risk for physical and mental health issues as a result of their occupation, as evidenced by the high rates of chronic non-communicable diseases in this population. The public safety system is divided into three sectors: the municipal guard, civilian police and military police. The latter group is associated with the highest morbidity and mortality, in addition to high rates of functional aging, burnout syndrome and poor sleep quality.^1-5^ Over time, officers who join the military police reduce their physical activity levels and change their eating habits.^6^ These changes contribute to the development of overweight/obesity and interfere with occupational activities that require physical effort.^7^

The physical fitness test is considered an essential tool for the assessment of physical fitness in military police officers.^8^ Internal regulations of the Paraná military police (Polícia Militar do Paraná) state that all officers must undergo the physical fitness test during the selection process. Before 2015, the only consequence of failing the test was the inability to access internal training programs;^9^ as a result, police officers or firefighters who opted not to take the courses offered by the institution would still be able to advance their careers without undergoing physical fitness testing. In December 2015, State Law No. 18.659, amending State Law No. 5.944/69 went into effect, and determined that approval on the physical fitness test should be considered a prerequisite for the promotion of military police officers.^10^

The aim of this study was therefore to evaluate whether the inclusion of the physical fitness test as a requirement for further promotions improved the physical fitness of military police officers in the state of Paraná, based on test results from 2016, 2017, 2018 and 2019 (practical implementation of the amendment insert by the State Law No. 18.659/2015).

## METHODS

This was a descriptive, longitudinal, comparative study (of temporal data) with intentional sampling. The results of the physical fitness tests conducted by the Paraná military police are published every year in a general report that is openly available to the public. These data were extracted and entered into Excel spreadsheets. The information was stratified by year (2016, 2017, 2018 and 2019) and occupation (public office), and included scores on the *shuttle run*, upper body strength (pull-ups, flexed-arm hang or push-ups) and Cooper test (12-minute run) as well as total sum scores across all three assessments. Military Police Law No. 076/2016 states that, to be considered fit for duty, officers must attain a total score of at least 150 across all three tests, considering the age of the individual. Scores are age-corrected based on a conversion table. Additionally, they must not have a score of zero on any of the tests; as such, if an officer earns a perfect score on two tests (for a sum total of 200) but a zero score on the third, they are deemed unfit.^11^

Statistical analyses were conducted using IBM SPSS, version 25.0 for Windows. Descriptive statistics were used to analyze all variables (*shuttle run*, upper body strength, 12-minute run and total score) in all four groups (2016, 2017, 2018, 2019). The Kolmogorov-Smirnov test was used to assess whether data were normally distributed (p > 0.05). Since this was not the case for some participant groups, comparisons were made using the Kruskal-Wallis H test, followed by Mann-Whitney U tests with Bonferroni correction to examine differences in the performance of military officers over the years. Effect sizes were calculated using the “r” statistic. Chi-square tests (χ^2^) were then used to verify whether the number of officers deemed fit for duty differed over the years. The level of statistical significance was set at 5%.

## RESULTS

The sample included 1,705 military police officers tested in 2016 (n = 103), 2017 (n = 664), 2018 (n = 410) and 2019 (n = 528). The results of these tests are shown in Table 1.

**Table 1:** Descriptive data for scores on different physical tests conducted from 2016 to 2019

Variables / Year	Mean ± SD	p-value	Post hoc (r)
*Shuttle run*			
2016	88.29 ± 20.75	< 0.001	
2017	95.64 ± 11.02		2016 < 2017 (0.17)
2018	96.56 ± 9.01		2016 < 2018 (0.23)
2019	96.91 ± 7.54		2016 < 2019 (0.21)
Upper body			
2016	63.05 ± 34.80	< 0.001	
2017	81.98 ± 26.90		2016 < 2017 (0.19)
2018	81.11 ± 26.75		2016 < 2018 (0.25)
2019	82.60 ± 24.81		2016 < 2019 (0.24)
12-minute run			
2016	42.34 ± 32.55	< 0.001	
2017	60.33 ± 28.55		2016 < 2017 (0.17)
2018	60.07 ± 28.38		2016 < 2018 (0.23)
2019	57.83 ± 28.53		2016 < 2019 (0.22)
Total score			
2016	193.27 ± 65.33	< 0.001	
2017	237.81 ± 48.99		2016 < 2017 (0.23)
2018	237.73 ± 48.81		2016 < 2018 (0.28)
2019	237.35 ± 46.64		2016 < 2019 (0.27)

r = effect size; SD = standard deviation.

Descriptive analysis showed that the best performance on the shuttle run test was observed in 2019, where the mean score was 96.91 (SD, 7.54) points. Similar findings were observed in the upper body strength test, where the highest score of 82.60 (SD, 24.81) was also recorded in 2019. The best performance in the 12-minute run test was recorded in 2017, where the mean score was 60.33 (SD, 28.38) points. Lastly, the highest total scores were observed in 2017, where the mean value was 237.81 (SD, 48.99) points.

**Figure 1:**
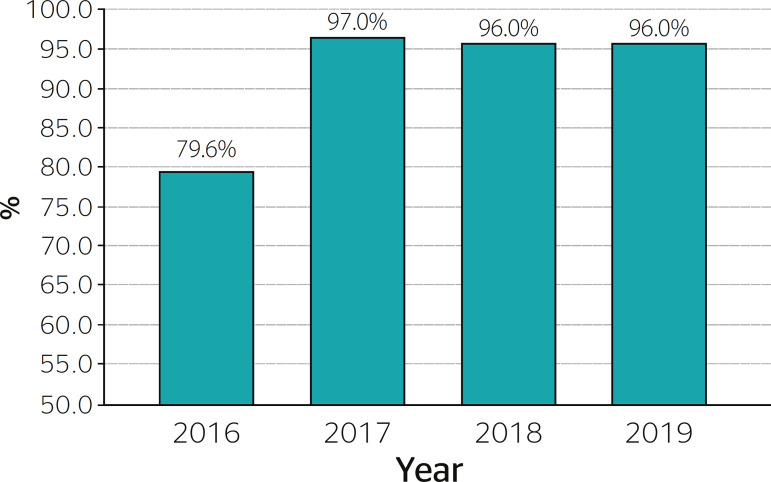
Percentage of military police officers who passed the physical fitness tests.

The Kruskal-Wallis test revealed that all variables changed significantly over the years (Table 1). The effect size (r) of these differences was classified as small. Figure 1 shows the percentage of military officers considered fit for duty based on their total scores across all three tests. The highest approval rate was observed in 2017 (97.0%) while the lowest was in 2016 (79.6%). Significant differences on this variable were observed between groups (χ^2^ = 64.713; p < 0.001).

## DISCUSSION

The aim of this study was to verify whether a new legal requirement could improve the physical fitness of state military officers. In a previous study of lifestyle risk factors in police officers in Recife, state of Pernambuco, 73% of the sample was found to be insufficiently active.^12^ From 2015 to 2017, in the Rio de Janeiro state alone, 475 military officers suffered nonfatal gunshot injuries.^13^ This population requires special attention due to their frequent exposure to excessive workloads, poor working conditions, high stress levels, risk of death, very poor sleep quality and lack of physical activity.^14-18^

One study^18^ of 258 military police officers in the state of Rio Grande do Sul identified a positive association between environmental quality of life and work rewards, while resilience was positively related to physical and psychological well-being, suggesting a possible protective effect on quality of life in this population. The study also showed that psychosocial stress and resilience can interfere with the quality of life of military officers, since all domains of the World Health Organization Quality of Life scale (WHOQOL-BREF) were significantly associated with resilience, as well as the effort, reward and overcommitment components of the effort-reward imbalance (ERI) model.^18^ While several studies in Brazil have examined the physical fitness of state military police officers based on their physical fitness test scores,^19-22^ few of these studies adopted longitudinal designs.

In an investigation conducted during Police Basic Training,^23^ military performance was assessed by a physical fitness test, in which 11.11% of the sample failed (n=16.549). Improved performance on the physical fitness test during training periods were also noted in recruits undergoing the first phase of the Sergeant Training Program offered by the Brazilian Military; according to a previous study, these individuals showed improvements in tests of upper body strength and resistance (push-ups and pull-ups) and 12-minute Cooper test.^24^ One commonality of these studies was the focus on tests conducted during preparation programs, when recruits are required to train regularly. Another longitudinal study,^25^ compared physical fitness tests completed at two time points: upon entering the 6-month preparation program and after 2 years and 7 months on the job. The study noted a decrease in cardiorespiratory fitness as well as muscle strength and resistance over time.^25^ By extending the assessment period beyond the duration of the training program, this investigation was able to address the limitation of the two aforementioned studies.

The present study focused on the effects of State Law No. 18.659/2015, which required state military officers to pass the physical fitness test in order to advance their careers; the fact that participants were required to pass the physical fitness test is the main difference between the third longitudinal study^25^ and the first two investigations cited.^23,24^ Figure 1 clearly shows that the proportion of military officers who passed the physical fitness test increased sharply in 2016, remaining high in subsequent years. The present findings are limited by the fact that the test results of officers engaged in operational vs administrative activities were not analyzed separately.

## CONCLUSIONS

The present study showed that the inclusion of the physical fitness test as a prerequisite for career advancement in 2016 was associated with improvements in the physical fitness of military police officers, and is likely to have played a causal role in that change. Future studies should continue to monitor the physical fitness test results obtained by military police officers in upcoming years, and replicate these comparisons in different sectors of the Paraná military police. Further research should also examine the physical fitness tests of military police units in different states, comparing those where physical fitness tests are required beyond the training period (as a prerequisite for promotions) and those where these assessments are only needed to access further training, and have no impact on career advancement.
